# Mitochondrial Hearing Loss: Genetic Variants and Clinical Progression

**DOI:** 10.7759/cureus.85825

**Published:** 2025-06-12

**Authors:** Toru Miwa, Kousuke Hashimoto, Toshiyuki Seto, Hirokazu Sakamoto

**Affiliations:** 1 Department of Otolaryngology, Teikyo University Hospital, Mizonokuchi, Kawasaki, JPN; 2 Department of Otolaryngology-Head and Neck Surgery, Kyoto University, Kyoto, JPN; 3 Department of Otolaryngology, Osaka Metropolitan University, Osaka, JPN; 4 Department of Clinical Genomics, Osaka Metropolitan University, Osaka, JPN; 5 Department of Auditory and Language Information Pathophysiology, Osaka Metropolitan University, Osaka, JPN

**Keywords:** heteroplasmy, m.3243a>g and m.1555a>g, mitochondrial dna mutations, phenotypic variability, sensorineural hearing loss

## Abstract

Mitochondrial diseases can affect multiple organ systems including the auditory pathway, leading to sensorineural hearing loss (SNHL). Although several mitochondrial DNA (mtDNA) mutations are linked to progressive hearing impairment, the underlying mechanisms and clinical course of mitochondrial hearing loss remain incompletely understood. In the present study, we analyzed the frequency and progression of mitochondrial mutations in 15 patients diagnosed with unexplained SNHL who underwent genetic testing at our institution. The most common mutations were m.3243A>G and m.1555A>G. Both are of particular interest due to their relatively high prevalence among mitochondrial mutations and strong clinical implications-m.3243A>G is linked to mitochondrial encephalomyopathy, lactic acidosis, and stroke-like episodes (MELAS) and diabetes, while m.1555A>G is associated with aminoglycoside-induced and non-syndromic hearing loss. Hearing loss associated with m.3243A>G is generally progressive, although the rate of deterioration varies among individuals. In contrast, the m.1555A>G cases remained stable throughout the follow-up period. No significant correlation was observed between the heteroplasmy levels and hearing deterioration, although a weak negative association was observed. Despite significant hearing impairment, hearing aids are underutilized by a considerable proportion of patients. These findings provide new insights into the phenotypic variability of mitochondrial hearing loss and underscore the need for longitudinal studies to assess its natural progression and potential therapeutic interventions.

## Introduction

Mitochondrial diseases arise from mutations in mitochondrial DNA (mtDNA), which encodes essential components of the oxidative phosphorylation system. Given the high metabolic demands of auditory sensory cells, the cochlea is particularly susceptible to mitochondrial dysfunction [1,2]. Consequently, many mitochondrial disorders manifest as primary or secondary hearing loss. Mitochondrial-related sensorineural hearing loss (SNHL) accounts for a notable clinical burden, with mutations like m.1555A>G found in about 1.7% of hearing loss cases [3]. Among individuals with mitochondrial diseases, up to 40.8% experience SNHL, underscoring the importance of mitochondrial dysfunction in auditory pathology [4].

Among the mtDNA mutations associated with hearing impairment, m.3243A>G and m.1555A>G are the most frequently documented [5]. The m.3243A>G mutation is associated with mitochondrial encephalomyopathy, lactic acidosis, and stroke-like episodes (MELAS) syndrome. Previous studies have demonstrated that this mutation leads to progressive SNHL, likely due to the degeneration of cochlear hair cells and spiral ganglion neurons [6]. Almost all patients with the m.3243A>G mutation experience a gradual decline in auditory function, which suggests sensory cell loss [7].

The m.1555A>G mutation is well known for its association with aminoglycoside-induced hearing loss, but can also cause non-syndromic SNHL in the absence of aminoglycoside exposure [8]. While some reports indicate that m.1555A>G may lead to slow, progressive hearing impairment, others suggest that it remains stable over time [8]. The phenotypic variability of this mutation raises questions regarding additional genetic (i.e., heteroplasmy) or environmental factors that may influence disease progression [9].

In this study, we investigated the prevalence and clinical course of mitochondrial mutations in patients with unexplained SNHL. By examining the relationship between heteroplasmy levels and hearing deterioration, we sought to provide further insights into the natural history of mitochondrial hearing loss and identify potential prognostic indicators for affected individuals. An expected clinical relevance of mitochondrial heteroplasmy is its influence on disease severity and symptom variability. In auditory disorders, higher heteroplasmy levels in cochlear cells may correlate with earlier onset or more rapid progression of hearing loss. A key hypothesis is that heteroplasmy thresholds determine mitochondrial dysfunction severity, affecting treatment response and prognosis. Understanding this relationship could lead to personalized therapies and better prediction of auditory outcomes in mitochondrial diseases.

## Materials and methods

Subjects

Fourteen Japanese individuals diagnosed with SNHL who underwent comprehensive genetic screening were enrolled in this study. The mtDNA mutations m.3243A>G, m.1555A>G, and m.7511T>C were detected in these individuals. The age of the cohort ranged from 15 to 55 years. Informed consent was obtained from all participants in accordance with ethical guidelines, and the study protocol was approved by the Ethics Review Committee, Osaka Metropolitan University (approval number: 2020-240).

Audiological assessment

Hearing acuity was quantified based on the pure-tone average (PTA) across six octave frequencies: 250 Hz, 500 Hz, 1000 Hz, 2000 Hz, 4000 Hz, and 8000 Hz. Serial audiological evaluations were performed using standardized pure-tone audiometry and speech discrimination tests to monitor the auditory function over time. Disease progression was assessed by computing the rate of change in PTA between the baseline and final evaluations.

Genetic and mutational analysis/quantification of heteroplasmy levels

Genomic and mitochondrial DNA were extracted from peripheral blood samples using standard phenol-chloroform extraction methods. Screening for mitochondrial DNA mutations (m.3243A>G, m.1555A>G, and m.7511T>C) and heteroplasmy ratios was outsourced to BML, Inc. (Tokyo, Japan) and Kazusa Genetic Laboratory (Chiba, Japan) and analyzed using the next-generation sequencing (NGS)/Invader method and Sanger sequencing. Briefly, NGS is a high-throughput method that allows simultaneous sequencing of millions of DNA fragments, enabling comprehensive analysis of multiple genes or entire genomes. The Invader method is a signal amplification technique used for detecting specific DNA mutations with high sensitivity and specificity, often in a targeted manner. Sanger sequencing is a traditional method that sequences DNA one fragment at a time using chain-terminating nucleotides, offering high accuracy but lower throughput than NGS.

Outcome measures

The primary outcome of interest was the longitudinal progression of hearing thresholds and speech discrimination scores. The secondary endpoints included mutation prevalence, clinical phenotypic variability, utilization of auditory prostheses, comorbid systemic conditions, and familial aggregation of hearing loss.

Statistical analysis

The Spearman's rank correlation coefficient was used to assess the association between the rate of hearing deterioration and heteroplasmy levels. Additionally, the correlations between changes in speech discrimination scores and heteroplasmy ratios were analyzed. Statistical significance was defined as p < 0.05.

## Results

Demographic information is shown in Table 1 and Table 2. Among the study population, 10 individuals were found to have the m.3243A>G mutation, with an average age of 36.4 ± 11.7 years and a male-to-female ratio of 4:7. The follow-up period was up to 14 years, with an average of 3.73 years. In this group, five (50%) of the patients had progressive hearing loss, while four (40%) presented with additional symptoms. Hearing aids (HAs) and cochlear implants (CIs) were used in seven (70%) of patients. All of the patients had good hearing when they used hearing devices. Other comorbidities, such as diabetes and cardiac conditions, were noted in five (50%) of cases. For the m.1555A>G mutation, three individuals were affected, with a mean age of 41.3 ± 22.8 years and a male-to-female ratio of 1:2. In this group, two (66.6%) had hearing loss, and one (33.3%) had additional auditory symptoms. One patient had unilateral hearing loss, but it was congenital and the details were unclear. HAs were used in one (33.3%). Systemic conditions, including neurological complications, were observed in two (66.6%) of cases.

**Table 1:** Demographic and clinical information of patients with mitochondrial or related hearing loss. It includes gene variants, heteroplasmy levels, age, sex, symptoms (e.g., HL, LiD, tinnitus), hearing devices used, and audiogram types. Family history of hearing loss or related diseases (e.g., DM) is noted, along with systemic complications such as cardiac or renal disorders. Occupation refers to the patient’s current or previous job. Variant2 lists additional nuclear gene variants. Audiogram descriptions indicate the pattern and severity of sensorineural hearing loss (SNHL). HL: hearing loss; LiD: listening difficulty; HA: hearing aid; CI: cochlear implant; DM: diabetes meillitus.

No	Gene	Variant1	Heteroplasmy	Age	Sex	Variant2	Symptoms	Hearing devices	Audiogram types	Family history	Complications	Occupation
1	MT-TL1	NC_012920.1 m.3243A>G	11.6	52	M	None	Progressive HL	HA	Bilateral moderate descending SNHL	Mother: DM/HL	DM, Cardiac hypertrophy	Sales position
2			12.0	47	F	GJB2 p.V37I hetero	Progressive HL	HA	Bilateral moderate flat SNHL	Father: HL, Mother: HL, Aunt: HL	Myopathy	Housewife
3			13.3	43	M	None	Progressive HL, Tinnitus	HA	Bilateral severe flat SNHL	Mother: DM/HL/Dementia	DM, HT	Unknown
4			19.7	55	F	None	HL	HA	Bilateral severe descending SNHL	None	Renal failure, cardiac disturbance	High school teacher
5			30.0	29	F	None	Progressive HL	None	Bilateral moderate descending SNHL	None	None	Unknown
6			35.0	31	F	None	HL, Vertigo	HA	Bilateral moderate flat SNHL	None	None	Unknown
7			39.6	29	F	None	HL	HA	Bilateral moderate flat SNHL	None	None	Game center staff
8			39.7	25	M	None	LiD	None	Bilateral moderate descending SNHL	None	None	Game center staff
9			41.0	24	F	None	LiD	None	Bilateral moderate descending SNHL	Mother: HL, Aunt: HL, Brother: DM/HL	None	Nursery teacher
10			45.7	29	M	None	Progressive HL	CI	Bilateral moderate flat SNHL	None	DM, HT, Cerebellar atrophy	Civil engineer
11	MT-RNR1	NC_012920 m.1555A>G	99.9	54	F	None	LiD, Tinnitus	None	Bilateral light descending SNHL	None	Hyperlipidemia, Migraine	Elementary school teacher
12			Unknown	15	F	GJB2 R12H hetero、TMPRSS3 F71S hetero	HL, Hyperacusis, Dizziness	HA	Unilateral moderate flat SNHL Unilateral deaf	None	Cerebellar atrophy	Unknown
13			Unknown	55	M	None	Progressive HL, Tinnitus	None	Bilateral severe high-tone descent type SNHL	Sister: HL	None	Unknown
14	MT-TS1	NC_012920.1 m.7511T>C	Unknown	54	F	None	Progressive HL, Tinnitus	HA	Bilateral severe flat SNHL	Mother: HL, Uncle: HL	DM, Cardiac sarcoidosis, Renal failure	Unknown

**Table 2:** Clinical characteristics of patients with three mitochondrial DNA variants. For each group, the number of cases (n), mean age ± SD, sex distribution, and proportions with systemic complications and family history are listed. Symptom frequencies include hearing loss (HL), listening difficulty (LiD), tinnitus, vertigo/dizziness, and hyperacusis. The proportion of patients using hearing aids or cochlear implants is also shown. Values are presented as counts and percentages. This comparison highlights phenotypic differences and shared features among patients with distinct mitochondrial mutations.

		m.3243A>G	m.1555A>G	m.7511T>C
n		10	3	1
Mean±SD(years)		36.4±11.7	41.3±22.8	54
Sex (M:F)		4:7	1:2	0:1
Complication +		10 (50.0%)	2 (66.6%)	1 (100%)
Family history +		4 (40.0%)	1 (33.3%)	1 (100%)
Symptoms	HL	8 (80.0%)	2 (66.6%)	1 (100%)
	LiD	2 (20.0%)	1 (33.3%)	0 (0%)
	Tinnitus	1 (10.0%)	2 (66.6%)	1 (100%)
	Vertigo/Dizziness	1 (10.0%)	1 (33.3%)	0 (0%)
	Hypeacusis	0 (0%)	1 (33.3%)	0 (0%)
Hearing aids/Cochlear implant +		7 (70.0%)	1 (33.3%)	1 (100%)

**Figure 1:**
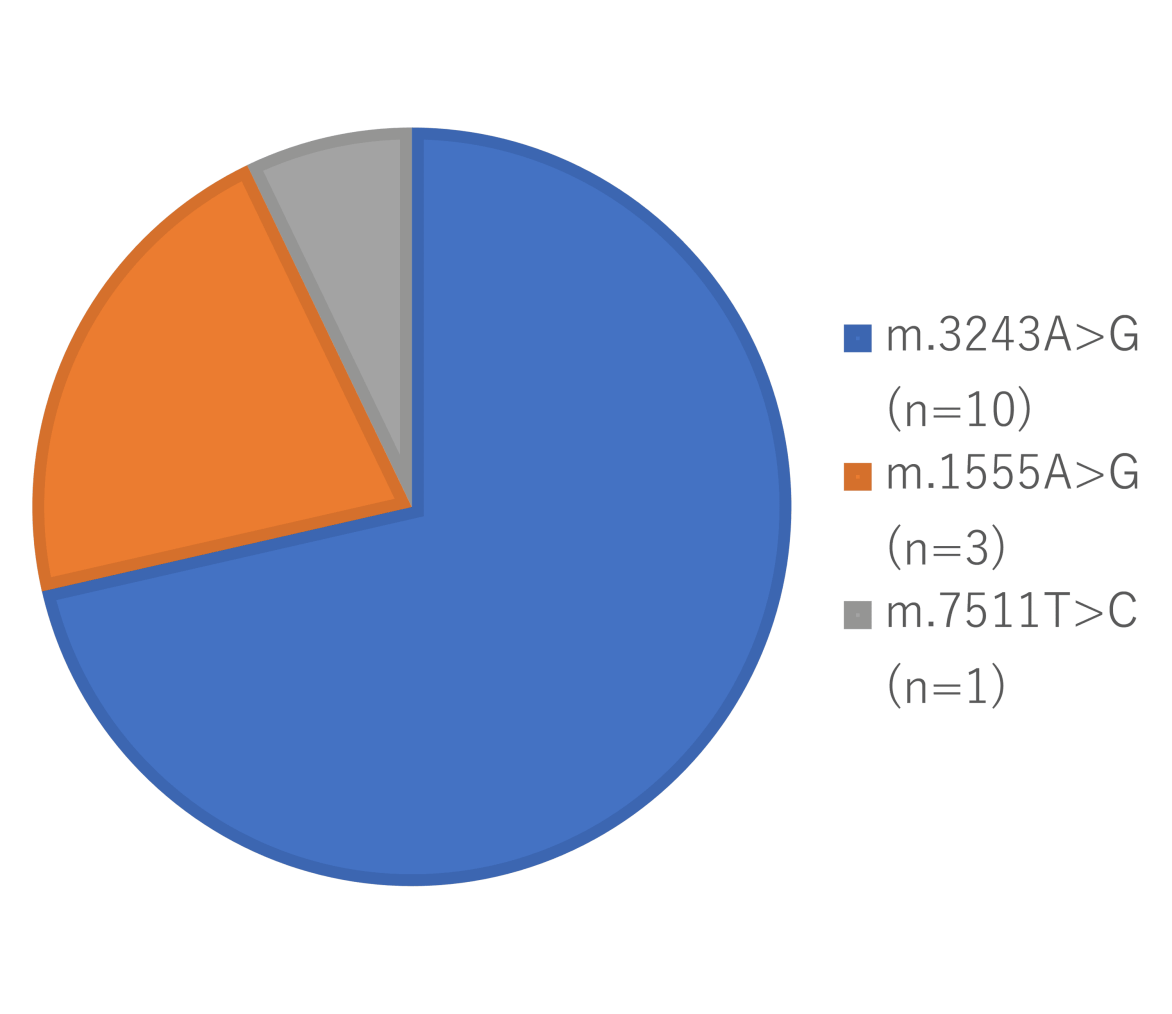
Proportion of patients harboring specific mtDNA mutations.

A single 54-year-old female patient was found to carry the m.7511T>C mutation, displaying a one (100%) prevalence of hearing loss, and using HAs. This mutation is associated with significant systemic comorbidities, including diabetes and cardiac sarcoidosis. The m.3243A>G mutation was the most prevalent mutation in our cohort, identified in 10 (72%) patients, which is consistent with its prevalence in broader epidemiological cohorts. The m.1555A>G mutation was detected in three (21%) patients, while one (7%) patient harbored the m.7511T>C mutation (Figure 1).

**Figure 2:**
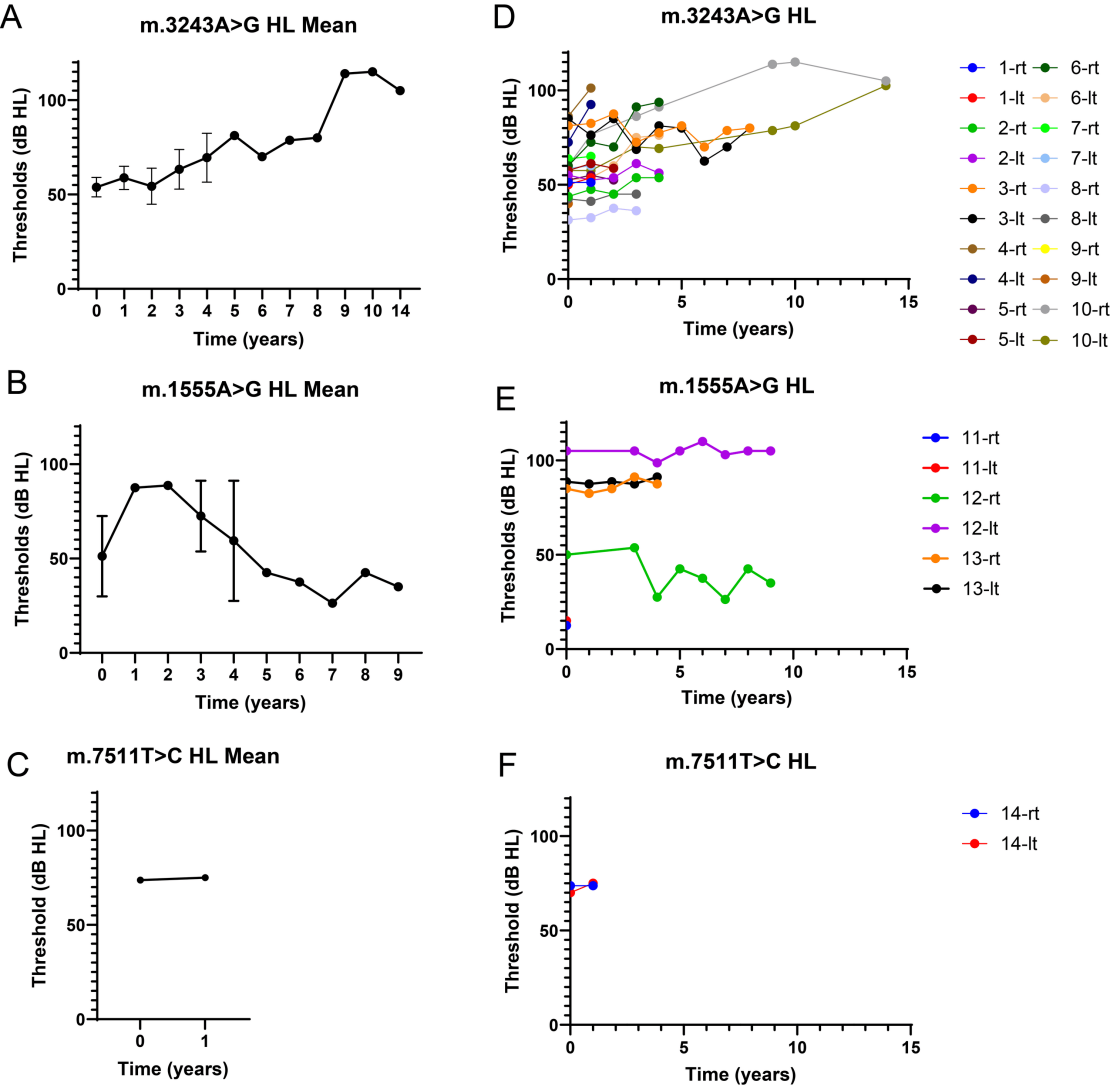
Course of hearing levels in each mutation (A, B, C) Mean of hearing thresholds in m.3243A>G (A), m.1555A>G (B), and m.7511T>C (C). (D, E, F) Spagetti plot in in m.3243A>G (D), m.1555A>G (E), and m.7511T>C (F).

**Figure 3:**
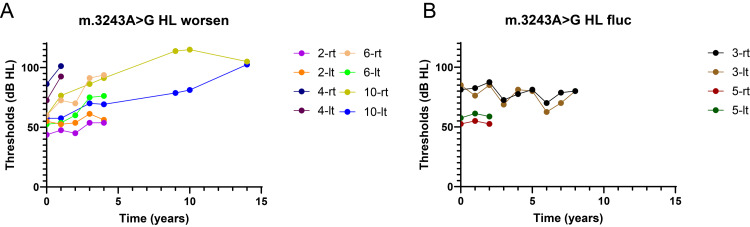
Data on patients in m.3243A>G with hearing changes (A, B) Spagetti plot with worsening (A) and fluctuating (B) conditions in m.3243A>G.

**Figure 4:**
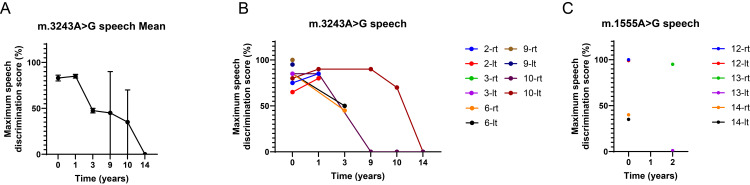
Course of speech discrimination (A) Mean of speech discrimination. (B, C) Spagetti plot in m.3243A>G (B), m.1555A>G (C).

The progression of hearing loss varies among the different genetic subtypes. In the m.3243A>G cases, hearing loss was generally progressive but with varying rates of deterioration (Figure 2A). Among the 20 ears assessed, eight (40%) ears exhibited a slow but steady decline in hearing threshold, four (20%) ears demonstrated fluctuating hearing levels, and the remaining patients showed stable hearing function during the study period (Figures 2D, 3A, 3B). Speech discrimination also progressively declined in most patients carrying this mutation (base mean speech discrimination: 83.0％, three years: 47.5%, 10 years: 35.0%, and 14 years: 0%, Figures 4A, 4B).

Conversely, patients with m.1555A>G mutation exhibited stable hearing throughout the follow-up period. Of the six ears evaluated in these patients, three showed an increase in the threshold from the first visit, and no further deterioration was observed. One ear exhibited fluctuating hearing (Figures 2B, 2E). As no cases of phonetic discrimination hearing tests were performed over time, it was not possible to follow the progress of the patients (Figure 4C).

In the case of the m.7511T>C mutation, hearing thresholds remained unchanged during the study period (Figures 2C, 2F).

**Figure 5:**
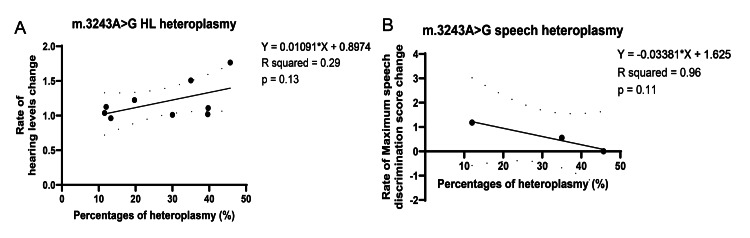
Correlation between hearing change rate and heteroplasmy (A) Correlation between rate of hearing levels change and percentage of heteroplasmy. (B) Correlation between rate of speech discrimination change and percentage of heteroplasmy.

A weak but non-significant correlation was noted between heteroplasmy levels and hearing progression in m.3243A>G cases (Figure 5A, p = 0.13). A negative, non-significant correlation was noted between heteroplasmy levels and the rate of change of speech discrimination in the m.3243A>G cases (Figure 5B, p = 0.11). This suggests that while heteroplasmy may contribute to disease severity, other factors, such as mitochondrial function in specific cochlear regions, may play a role in determining clinical outcomes.

## Discussion

This study contributes to the growing body of evidence regarding mitochondrial hearing loss, particularly m.3243A>G, a well-characterized mutation associated with progressive SNHL. The observed gradual decline in auditory function in m.3243A>G carriers suggests a degenerative process involving both cochlear hair cells and spiral ganglion neurons. The variability in progression rates across affected individuals underscores the interplay between genetic, environmental, and physiological modulators that determine disease severity. Given the heteroplasmic nature of mitochondrial DNA mutations, the tissue-specific distribution of mutated mitochondria within the cochlear structures is likely a critical determinant of individual disease trajectories. A notable finding of this study was the presence of fluctuating auditory thresholds in some m.3243A>G carriers, suggesting that mitochondrial dysfunction extends beyond primary neurodegeneration. The involvement of secondary cochlear structures such as the stria vascularis and spiral ligament may have contributed to this phenomenon [10,11]. Dysregulation of metabolic stress and ion homeostasis, consequences of mitochondrial impairment, may explain these fluctuations. This raises the possibility that metabolic-targeted therapies, such as cochlear vasodilators, antioxidants, or mitochondrial protective agents, may provide therapeutic benefits to specific m.3243A>G subpopulations [12-14]. Further studies are warranted to identify subgroups of patients who may derive clinical benefits from such interventions.

In contrast, m.1555A>G was associated with relatively stable auditory thresholds in our cohort, diverging from previous reports suggesting progressive hearing loss in some carriers [5,9]. While m.1555A>G is a well-established risk factor for aminoglycoside-induced ototoxicity, spontaneous auditory decline in its absence remains variable. The observed stability may reflect the influence of modifying genetic factors, environmental exposure, or the limitations of the study’s follow-up duration. Nuclear-encoding genes that regulate mitochondrial biogenesis and protein synthesis have been implicated in the modulation of m.1555A>G phenotypic expression [9]. Additionally, mitochondrial haplogroup variations have been associated with differential penetrance of mitochondrial disorders [9]. These findings highlight the need for future investigations into mitochondrial-nuclear genomic interactions to refine the risk stratification for progressive hearing loss in m.1555A>G carriers.

Despite previous evidence suggesting a correlation between heteroplasmy levels and disease severity [15], our data do not support this association in the context of hearing loss. This discrepancy may arise from tissue-specific heteroplasmy differences, as blood-derived measures may not accurately represent the cochlear mitochondrial content [16,17]. Given the logistical challenges of direct cochlear tissue sampling in living patients, future efforts should focus on the development of novel biomarkers, such as mitochondrial RNA expression profiles or noninvasive imaging modalities, to improve the precision of mitochondrial dysfunction assessment in auditory pathology.

A clinically significant observation in our study was the underutilization of hearing aids among the affected individuals. Despite significant auditory deficits, many m.3243A>G and m.1555A>G carriers did not adopt amplification devices. This reluctance may stem from multiple factors including limited awareness, social stigma, and barriers to audiological care. In addition, fluctuating hearing thresholds may contribute to skepticism regarding the benefits of amplification. Given the progressive nature of mitochondrial SNHL, early hearing aid adoption may be crucial for preserving the auditory processing pathways and optimizing long-term communication outcomes. Future research should evaluate the impact of early hearing intervention strategies, including cochlear implantation, on the auditory and quality-of-life outcomes in this patient population.

Given the irreversible nature of mitochondrial dysfunction in many cases, emerging therapeutic strategies should aim to mitigate disease progression. Mitochondria-targeted antioxidants, including coenzyme Q10, alpha-lipoic acid, and mitoquinone, have shown promise in preclinical models and warrant further clinical evaluation [18]. Additionally, advances in mitochondrial gene therapy, including mitochondrial base editing and transplantation, represent potential avenues for future therapeutic developments [19]. Although these approaches are experimental, they offer a foundation for the development of precision medicine strategies for mitochondrial SNHL.

This study has several limitations that must be acknowledged when interpreting the findings. The primary constraint is the relatively small sample size, which limits the statistical power and generalizability of the conclusions. This study lacks multivariate analysis, and only a simple correlation coefficient (Spearman) was used, without taking into account other confounding factors such as age, comorbidities, or nuclear gene mutations. With only 15 participants, the subtle associations between mitochondrial mutations, heteroplasmy levels, and auditory outcomes may not have been adequately captured. Larger multicenter studies are necessary to validate these results and identify additional modifying factors that influence disease progression. Among patients with m.1555A>G, hyperlipidemia, migraine, and cerebellar atrophy were reported as complications, but it is difficult to determine the relationship between these conditions, and they cannot be excluded as complications. In addition, the retrospective nature of this study introduced a potential bias in data collection and interpretation. The reliance on previously recorded clinical and audiometric data introduces variability in the follow-up intervals and testing methodologies, which could affect the robustness of our conclusions. A prospective longitudinal design with standardized audiometric protocols would provide a more rigorous assessment of mitochondrial hearing loss progression and its correlation with heteroplasmy levels. Finally, cochlear implantation outcomes in patients with mitochondrial SNHL were not analyzed in this study. Although cochlear implantation is a viable intervention for severe-to-profound SNHL, limited data exist regarding its efficacy, speech perception outcomes, and long-term neural plasticity adaptations in individuals with mitochondrial mutations [20]. Future studies should explore whether heteroplasmy levels influence implant success and auditory rehabilitation in this patient population.

## Conclusions

This study provides important insights into the clinical diversity and progression patterns of mitochondrial hearing loss, with a particular focus on the m.3243A>G and m.1555A>G mutations. Our findings demonstrate that hearing loss in individuals carrying the m.3243A>G mutation tends to be progressive, albeit with variable trajectories, ranging from stable to rapidly deteriorating, and in some cases, fluctuating thresholds. In contrast, patients with the m.1555A>G mutation often exhibit stable hearing over time, reinforcing the idea that not all mitochondrial mutations follow a predictable clinical course. Despite significant hearing impairment, many affected individuals underutilize hearing aids, pointing to a gap in hearing rehabilitation practices. This is thought to be due to insufficient patient education and counseling. The lack of a strong correlation between heteroplasmy levels and auditory decline suggests that other factors-such as cochlear mitochondrial distribution or environmental influences-may play a more prominent role in determining disease severity. These observations highlight the complexity of mitochondrial pathophysiology and underscore the importance of individualized monitoring and management strategies. Future research should focus on identifying reliable biomarkers, exploring new treatments, and clarifying the role of mitochondrial dysfunction in hearing loss. Key improvements include conducting multicenter, prospective cohort studies with standardized screening, assessing environmental and lifestyle factors, and evaluating quality of life and hearing aid satisfaction. Using mitochondrial tissue samples from the ear or developing alternative biomarkers is also recommended.
